# Slow Clearance of *Plasmodium falciparum* in Severe Pediatric Malaria, Uganda, 2011–2013

**DOI:** 10.3201/eid2107.150213

**Published:** 2015-07

**Authors:** Michael Hawkes, Andrea L. Conroy, Robert O. Opoka, Sophie Namasopo, Kathleen Zhong, W. Conrad Liles, Chandy C. John, Kevin C. Kain

**Affiliations:** University of Alberta, Edmonton, Alberta, Canada (M. Hawkes);; University of Toronto, Toronto, Ontario, Canada (M. Hawkes, A.L. Conroy, K.C. Kain);; Mulago Hospital, Kampala, Uganda (R.O. Opoka); Makerere University, Kampala (R. Opoka);; Jinja Regional Referral Hospital, Jinja, Uganda (S. Namasopo);; University Health Network–Toronto General Hospital, Toronto (K. Zhong, K.C. Kain);; University of Washington, Seattle, Washington, USA (W.C. Liles);; University of Minnesota, St. Paul, Minnesota, USA (C.C. John);; Sandra Rotman Centre for Global Health, Toronto (K.C. Kain)

**Keywords:** malaria, artesunate, resistance, child, single nucleotide polymorphism, kelch 13 propeller domain, parasites, Plasmodium falciparum, Uganda

## Abstract

*Plasmodium falciparum* resistance to artemisinin derivatives is emerging in Asia. We examined molecular markers of resistance in 78 children in Uganda who had severe malaria and were treated with intravenous artesunate. We observed in the K13-propeller domain, A578S, a low-frequency (3/78), nonsynonymous, single-nucleotide polymorphism associated with prolonged parasite clearance.

Resistance of *Plasmodium falciparum* parasites to artemisinin derivatives threatens the current first-line treatment for severe malaria. Artemisinin resistance was first reported in 2009 in Pailin, western Cambodia (*1*), and has since become prevalent in the greater Mekong Delta, Vietnam, where standard 3-day courses of artemisinin combination therapies for uncomplicated *P.* *falciparum* malaria are now failing (*2*–*4*).

Among several putative genetic determinants of parasite resistance to artesunate (*3*,*5*), polymorphisms in the propeller domain of a *kelch* gene on chromosome 13 (*PF3D7_1343700*; K13) are now recognized as the major determinant of artemisinin resistance observed in *P. falciparum* isolates from patients in Southeast Asia (*3*,*4*,*6*,*7*). Various single amino acid substitutions in the K13 protein are associated with a mean increase of 116% in the parasite clearance half-life (t_1/2_) (*4*). The mechanism of resistance has been illuminated by a recent study of the *P. falciparum* transcriptomes from >1,000 acute malaria episodes (*6*). Slow-clearing parasites exhibited increased expression of unfolded protein response pathways (e.g., chaperone complexes); these pathways may mitigate protein damage caused by artemisinin. Slow-clearing parasites also exhibited decreased expression of proteins involved in DNA replication and decelerated development at the young ring stage. Haplotype analysis suggests that K13 mutations emerged independently in multiple geographic locations in Southeast Asia, causing concerns about the ability to contain resistant parasites (*7*).

With the widespread use of artemisinin treatment, resulting in continued pressure for natural selection of the most resistant parasites, resistance may emerge in regions beyond Asia, including Africa. The possible increase of parasite resistance to treatment highlights an urgent need to map K13 mutations throughout the malaria-endemic world (*7*). Consequently, recent molecular epidemiologic analyses of K13 in Senegal (*8*) and Uganda (*9*) and in a large collection of >1,100 infections from sub-Saharan Africa (*10*) have been undertaken, revealing the absence of nonsynonymous single-nucleotide polymorphisms (SNPs) associated with artemisinin resistance in Southeast Asia. Other distinct nonsynonymous SNPs have been discovered in parasites of African origin (*9*,*10*), but association of these mutations with a resistance phenotype has not been shown.

## The Study

We examined parasite clearance kinetics and sequenced the parasite K13 gene in a cohort of 78 children with severe malaria (including 8 children who died) from the placebo group (being treated with artesunate alone) of a randomized, controlled trial conducted at the Jinja Regional Referral Hospital, Uganda, during July 12, 2011–June 14, 2013 (*11*). Inclusion and exclusion criteria have been described elsewhere (*11*). The median age of patients was 2.0 years (range 1.0–8.0 years), and 38 (49%) were female. All patients were treated intravenously with artesunate (Guilin Pharmaceutical, Shanghai, China) prequalified by the World Health Organization and according to the Organization’s guidelines (*12*).

Giemsa-stained peripheral blood smears (thin and thick) were assessed for quantitative malaria parasite density by light microscopy at a quality-controlled central research laboratory, the Makerere University–Johns Hopkins University Research Collaboration Core Laboratory, which is certified by the College of American Pathologists. For each patient, 5 serial parasite densities were measured according to the following sampling schedule: 1) admission; 2) ≈12 hours later; 3) morning of the second day of admission; 4) morning of the third day of admission; and 5) morning of the fourth day of admission.

To measure parasite clearance kinetics, we used a standardized tool, the parasite clearance estimator, which expresses parasite clearance as the parasite’s half-life (t_1/2_), which was calculated by using the slope of the linear portion of the curve of log-transformed parasite densities over time (*13*). In addition, we computed the parasite clearance time, defined as the interval between the start of treatment and the first of 2 sequential negative peripheral blood films (*14*).

To determine molecular markers of resistance, we amplified and sequenced the *PF3D7_1343700* gene by using the nested PCR method, as described (*3*), with some modifications. DNA was extracted from cryopreserved erythrocyte fractions by using QIAGEN columns (QIAGEN, Valencia, CA, USA) according to the manufacturer’s instructions. The K13-propeller domain was amplified by using K13-1 5′-CGGAGTGACCAAATCTGGGA-3′ and 5′-K13-4 GGGAATCTGGTGGTAACAGC-3′ for the primary PCR and K13-2 5′-GCCAAGCTGCCATTCATTTG-3′ and K13-3 5′-GCCTTGTTGAAAGAAGCAGA-3′ for the nested PCR. DNA sequencing of the 810-bp nested PCR product was performed to determine the amino acid haplotype of residues. Results were aligned to reference PF3D7 kelch protein, putative (PF13_0238) mRNA, complete coding sequence (National Center for Biotechnology Information reference sequence XM_001350122.1).

We identified limited diversity within the K13 gene. For 16 loci tested (amino acid positions 439, 441, 458, 465, 467, 476, 493, 522, 539, 543, 557, 558, 580, 617, 619, and 637), the wild-type sequence was found in all 78 parasite amplicons. We did not observe any of the most common amino acid substitutions in K13 associated with artemisinin resistance in *P. falciparum* isolates from Cambodia (C580Y, R539T or Y493H) (*3*), nor the I543T and N458Y mutations most strongly associated with increased clearance t_1/2_ in another recent study (*4*), nor the M476I mutation selected in vitro under artemisinin pressure (*3*). Similarly, these point mutations were absent in isolates from Senegal and Uganda and in >1,100 *P. falciparum* parasites from 14 sites across sub-Saharan Africa (*8*–*10*). However, a previously reported point mutation, A578S (*9*,*15*), was found in 3 (3.8%) of 78 infections. 

The Table shows infections with A578S parasites compared with infections caused by wild-type parasites. Parasite clearance time was prolonged in infections with A578S mutant parasites, and a similar trend was observed for clearance t_1/2._ These 2 types of infections showed no differences in prior artemisinin exposure, number of deaths, or recrudescence or reinfection at day 14 from date of admission.

**Table:** Characteristics of children infected with severe *Plasmodium falciparum* malaria parasites harboring the Kelch 13 A578S polymorphism compared with children infected with wild-type parasites, Uganda

Characteristic	A578S mutation, n = 3	Wild-type parasite, n = 75	p value
Parasite clearance time, median (interquartile range), h	80 (71–200)	45 (40–64)*	0.033
Clearance half-life, median, (interquartile range), h	5.9 (5.6–10.7)	4.5 (3.6–5.8)*	0.074
Prior artemisinin exposure, no. (%)	1 (33.3)	19 (25.3)	1.000
Deaths, no. (%)	0	8 (10.7)	1.000
Recrudescence or reinfection	0	0	1.000

*For 7 patients who died, parasite clearance time could not be calculated because of incomplete parasite clearance prior to death. For these same 7 children, the parasite clearance half-time (t_1/2)_ could not be calculated because of insufficient data points. For 1 child who died, clearance of parasitemia before death was documented; thus, the parasite clearance time and t_1/2_ could be computed. Estimates of parasite clearance time and clearance t_1/2_ are based on the remaining 68 patients. All fatal cases were associated with wild-type parasites.

## Conclusions

The role of the A578S amino acid substitution is unclear, but it occurs near the most common K13-propeller mutation (C580Y), which has been associated with delayed parasite clearance in Southeast Asia and with tolerance to artemisinin in vitro (*3*). Computational modeling suggests that A578S should considerably affect the tertiary structure of the K13 protein, thereby destabilizing the domain scaffold and altering its function (*15*). Isolates with A578S exhibited a phenotype of prolonged clearance under artesunate treatment in our study. Delayed clearance of A578S parasites was not observed in previous reports (*4*,*9*), although the number of isolates in our study and in others was small. Because multiple independent mutations in K13 have arisen in geographic regions engaged in intense treatment of malaria with artemisinin derivatives (*7*) and because only 2 point mutations were necessary to confer drug tolerance in vitro to a *P. falciparum* isolate from Tanzania (*3*), we are concerned that parasites with the A578S mutation are already causing severe malaria in children in Uganda, although with low frequency.

The nonsynonymous SNP A578S in the K13-propeller domain may represent another putative marker of delayed response to artesunate, although this indicator occurred infrequently in our cohort of children with severe malaria in Uganda. Our study documents an association between A578S and prolonged parasite clearance time, a finding relevant for future monitoring of *P. falciparum* response to parenteral artesunate in children in Africa.
